# Spread of *bla*_CTX-M-9_ and Other Clinically Relevant Resistance Genes, Such as *mcr-9* and *qnrA1*, Driven by IncHI2-ST1 Plasmids in Clinical Isolates of Monophasic *Salmonella enterica* Serovar Typhimurium ST34

**DOI:** 10.3390/antibiotics12030547

**Published:** 2023-03-09

**Authors:** Xenia Vázquez, Javier Fernández, Miriam Alkorta, María de Toro, M. Rosario Rodicio, Rosaura Rodicio

**Affiliations:** 1 Departamento de Biología Funcional, Área de Microbiología, Universidad de Oviedo (UO), 33006 Oviedo, Spain; 2 Grupo de Microbiología Traslacional, Instituto de Investigación Sanitaria del Principado de Asturias (ISPA), 33011 Oviedo, Spain; 3 Servicio de Microbiología, Hospital Universitario Central de Asturias (HUCA), 33011 Oviedo, Spain; 4 Centro de Investigación Biomédica en Red-Enfermedades Respiratorias, 30627 Madrid, Spain; 5 Research & Innovation, Artificial Intelligence and Statistical Department, Pragmatech AI Solutions, 33001 Oviedo, Spain; 6 Servicio de Microbiología, Hospital Universitario de Donostia (HUD)-IIS Biodonostia, 20014 San Sebastián, Spain; 7 Plataforma de Genómica y Bioinformática, Centro de Investigación Biomédica de La Rioja (CIBIR), 26006 Logroño, Spain; 8 Departamento de Bioquímica y Biología Molecular, Universidad de Oviedo (UO), 33006 Oviedo, Spain

**Keywords:** antimicrobial drug resistance, *Salmonella* genomic island 4, RR chromosomal resistance region, IncHI2, IS*CR*, complex class 1 integron, *bla*_CTX-M-9_, *qnrA1*, *mcr-9*

## Abstract

The monophasic 4,[5],12:i:-variant of *Salmonella enterica* serovar Typhimurium with sequence type ST34 has become one of the most prevalent non-typhoidal salmonellae worldwide. In the present study, we thoroughly characterized seven isolates of this variant detected in a Spanish hospital and selected based on cefotaxime resistance and cefoxitin susceptibility, mediated by *bla*_CTX-M-9_. For this, conventional microbiological techniques, together with whole genome sequencing performed with the Illumina platform, were applied. All selected isolates carried the resistance region RR or variants therein, and most also contained the SGI-4 genomic island. These chromosomal elements, typically associated with monophasic *S*. Typhimurium ST34, confer resistance to traditional antibiotics (ampicillin, streptomycin, sulfonamides, and tetracycline) and tolerance to heavy metals (mercury, silver, and copper). In addition, each isolate carried a large IncHI2-ST1 conjugative plasmid containing additional or redundant resistance genes. All harbored the *bla*_CTX-M-9_ gene responsible for cefotaxime resistance, whereas the *qnrA1* gene mediating fluoroquinolone resistance was detected in two of the plasmids. These genes were embedded in IS*CR1*-bearing complex class 1 integrons, specifically In60-like and In36-like. The *mcr-9* gene was present in all but one of the IncHI2-ST1 plasmids found in the analyzed isolates, which were nevertheless susceptible to colistin. Most of the resistance genes of plasmid origin clustered within a highly complex and variable region. The observed diversity results in a wide range of resistance phenotypes, enabling bacterial adaptation to selective pressure posed by the use of antimicrobials.

## 1. Introduction

Non-typhoid serotypes of *Salmonella enterica* (NTS) are among the main causes of food-borne human infections worldwide. In 2020, salmonellosis was the second most frequent bacterial zoonosis in the European Union (EU), with 52,702 confirmed cases, being only outnumbered by campylobacteriosis [1]. NTS mostly causes self-limited infections that resolve in a few days and do not require antibiotic treatment. However, it can also produce more serious extra-intestinal infections, including bacteremia and sepsis, which mainly affect children, the elderly, and immuno-compromised persons. Third-generation cephalosporins and fluoroquinolones are first-line antibiotics for the treatment of such complicated cases of salmonellosis, with the latter not applicable to children [2]. Fortunately, resistance to these compounds, listed by the World Health Organization (WHO) as “critically important antimicrobials” with the highest priority for human medicine [3], remains low in *Salmonella* [4].

Resistance to broad-spectrum cephalosporins can be mediated by the production of extended-spectrum β-lactamases (ESBL) or AmpC β-lactamases. ESBLs can hydrolyze oxyimino-cephalosporins (third and fourth-generation cephalosporins) and monobactams, but not cephamycins or carbapenems, and are usually susceptible to β-lactamase inhibitors [5,6]. The majority of ESBLs belong to Ambler class A and include the TEM-, SHV-, and CTX-M-types, with the latter most frequently found in species of the order Enterobacterales. In addition to bacterial clonal expansion, plasmids of different incompatibility (Inc) groups, which have been regarded as epidemic, are contributing to the spread of ESBL-encoding genes, such as *bla*_CTX-M_ variants [5]. The acquisition of *bla*_CTX-M_ genes by these plasmids was shown to be mediated by several genetic elements, including IS*Ecp1*-like and IS*CR1*-like (Common Region) elements, both capable of mobilizing adjacent resistance genes [7,8]. Bacterial isolates harboring such plasmids pose a risk to human health and must be subjected to epidemiological surveillance.

In the present study, we report seven isolates of *S. enterica,* which have the *bla*_CTX-M-9_ gene on epidemic IncHI2 plasmids. They were recovered at a Spanish hospital from patients with gastroenteritis and assigned to monophasic *S*. Typhimurium with sequence type ST34, which has emerged as an important food-borne pathogen worldwide [9,10,11,12]. The epidemiological success of this pathogen has been at least in part attributed to the acquisition of two chromosomal genetic elements, the *Salmonella* genomic island SGI-4, an integrative and conjugative element (ICE) which carries genes for tolerance to copper/silver and arsenic compounds [13,14,15,16], and the so-called resistance region RR. The latter usually contains the *bla*_TEM-1B_, *strA*-*strB*, *sul2*, and *tet*(B) genes (for resistance to ampicillin, streptomycin, sulfonamides, and tetracycline), together with a mercury resistance locus, and can consist of one or two modules [17,18,19]. However, numerous variants of this region have been found, probably due to its intrinsic instability derived from the presence of multiple copies of IS*26* [14,20,21]. In addition to antibiotic and biocide resistance genes supplied by these chromosomal elements, the CTX-M-9 producers selected for the present study also carried plasmid-mediated colistin resistance (PMCR) genes (*mcr*) and plasmid-mediated quinolone resistance (PMQR) genes (*qnr*), as well as many other resistant determinants, further aggravating the problem.

## 2. Materials and Methods

### 2.1. Isolates and Antimicrobial Susceptibility Testing

Seven monophasic isolates of *S*. Typhimurium were characterized in the present study (Table 1). Their selection was based on resistance to cefotaxime (MIC ranging from 16 to >40 mg/L) and susceptibility to cefoxitin (MIC ≤4 mg/L), a phenotype shown to be associated with CTX-M-9 production. They were recovered from fecal samples of different patients attending the “Hospital Universitario de Donostia” (HUD), San Sebastián, Basque Country, Spain, between 2012 and 2016. Details on the age and sex of the patients are given in Table 1.

Antimicrobial susceptibility testing was performed with VITEK^®^2 (bioMérieux, Marcy-l’Étoile, France) for all *S. enterica* isolates detected at the hospital during the 2012–2018 period, and complemented with disk diffusion assays using commercially available discs (Oxoid, Basingstoke, UK) for those selected for the present study. MICs of the latter to ciprofloxacin were also determined by E-test (bioMérieux), and MICs to colistin by broth microdilution as recommended by EUCAST (www.eucast.es, accessed on 15 November 2022). In addition, the pefloxacin disk diffusion assay proposed by EUCAST was used as a surrogate of ciprofloxacin to screen for clinical fluoroquinolone resistance. Results were interpreted according to EUCAST clinical breakpoints v. 13 (https://www.eucast.org/clinical_breakpoints, accessed on 28 November 2022).

### 2.2. Whole Genome Sequencing and Bioinformatics Analysis

For genome sequencing, the total DNA of the seven isolates was extracted from overnight cultures grown in LB broth using the GenEluteTM Bacterial Genomic DNA Kit, following the supplier’s instructions (Sigma-Aldrich-Merck, Darmstadt, Germany). Sequencing was performed at the CIBIR (Centro de Investigación Biomédica, La Rioja, Spain) with Illumina technology. Fragment libraries of ca. 500 bp were prepared with the TruSeq PCR-free DNA Sample Preparation Kit. Paired-end reads of 100 or 150 nt were generated from the libraries in a HiSeq 1500 system. Genome reconstructions were performed with the online version of PLACNETw (https://castillo.dicom.unican.es/upload/, accessed on 23 June 2021), which assembles the reads by means of Velvet Optimizer and assigns the generated contigs to either the chromosome or to plasmids based on BLAST searches and contig coverage [22]. Information regarding the quality of the assemblies is compiled in Table S1. The genomes were deposited in GenBank under the accession numbers shown in Table S1 and below. They were annotated by the NCBI Prokaryotic Genome Annotation Pipeline (PGAP; https://www.ncbi.nlm.nih.gov/genome/annotation_prok/, accessed on 4 August 2021).

Bioinformatics analysis of the genomes were performed with the PLACNETw interface in conjunction with several tools available at the Center for Genomic Epidemiology (https://cge.cbs.dtu.dk/services/, last accessed on 16 February 2023), of the Technical University of Copenhagen (DTU), including SeqSero, MLST (multi-locus sequencing typing), CSIPhylogeny, ResFinder, PlasmidFinder, and pDLST (plasmid double locus sequence typing) for the IncHI2 plasmids characterized in the present study. Plasmid replicons were also identified by PLACNETw. The genetic context of the resistance genes, including *bla*_CTX-M-9_, *mcr-9,* and *qnrA1*, was established by joining relevant contigs using overlapping PCR reactions with specific primers, followed by Sanger sequencing (performed at STAB VIDA, Caparica, Portugal). The same strategy was used to determine the structures of the RR elements and their location within the bacterial chromosome and to generate the entire nucleotide sequence of the IncHI2 plasmid found in HUD 1/16 (pHUD 1/16), which harbors the highest number of resistance genes. Sequences of oligonucleotides used as primers in these approaches are shown in Table S2. Graphic representations derived from sequence information were generated with BRIG (Blast Ring Image Generator; https://brig.sourceforge.net/v0.95, accessed on 24 January 2023) or Easyfig BLASTn (https://mjsull.github.io/Easyfig/, accessed on 30 January 2023).

### 2.3. Conjugation Experiments

These experiments were performed using each of the ESBL producers as donors and *Escherichia coli* K-12 J53 resistant to rifampicin as the recipient. Specifically, 100 µL of the donor and 200 µL of the recipient, cultured overnight at 37 °C, were added to 1 mL Luria-Bertani (LB) liquid medium. The mixtures were maintained overnight at 28 °C and 37 °C without shaking. Transconjugants were selected on eosin–methylene blue agar (Oxoid) containing cefotaxime (8 mg/L) and rifampicin (50 mg/L). Two transconjugants per mating were tested for antimicrobial susceptibility, and the presence of the IncHI2 replicon was demonstrated by PBRT (PCR-Based Replicon Typing; [23]). The frequencies of plasmid transfer were calculated as the number of transconjugants per donor cell, with values corresponding to the average of two independent experiments.

### 2.4. Nucleotide Sequence Accession Numbers

The nucleotide sequences of HUD 1/12, HUD 2/12, HUD 3/12, HUD 2/14, HUD 3/15, HUD 1/16 and HUD 2/16 were deposited at the GenBank database under the accession numbers JAICCN000000000, JAICCM000000000, JAICCL000000000, JAICCK000000000, JAICCJ000000000, JAICCI000000000, JAICCH000000000, respectively (also shown in Table S1).

## 3. Results and Discussion

### 3.1. General Characteristics of the Isolates

During 2012–2018, 1865 isolates of *S. enterica* were detected at the HUD, but only 12 were resistant to broad-spectrum cephalosporins (cefotaxime or cefotaxime and cefoxitin). Thus, the frequency of such isolates was very low (0.64%), in line with information available for *S. enterica* associated with human cases in the EU (0.8%) [4]. For the present study, seven HUD isolates that were resistant to cefotaxime, but susceptible to cefoxitin, due to CTX-M-9 production were thoroughly characterized (Table 1).

The draft genomes of these isolates consisted of a total of 96 to 131 contigs (32 to 52 larger than 1 kb), with assembly sizes ranging from ca. 5122–5324 Mb (Table S1). Their antigenic formula, as determined in silico by SeqSero, was 4:i:-, and they were assigned to ST34 by MLST, also performed in silico. The number of SNP differences between genomes, as determined by CSIPhylogeny (Table S3), ranged from 3 or 5 up to 406. Therefore, some of the isolates were very closely related, even if they were recovered from different patients at different years, while others were distantly related.

Plasmids (one up to three) were found in all isolates. They belonged to incompatibility group IncH12 and were ColE or had a non-identified replicon (Table 1). The IncHI2 plasmids, found in all isolates, were assigned to ST1 by pDLST [24]. This scheme identified the *smr0018* and *srm0199* loci, with the former appearing twice in the plasmid of one of the isolates (HUD 3/15). As previously reported from other screens [10], the virulence plasmid pSLT, specifically of serovar S. Typhimurium, was not detected in any of the isolates under study.

### 3.2. Resistance Properties and Genetic Basis of Antimicrobial Drug Resistance

The monophasic *S*. Typhimurium ST34 isolates analyzed in the present study displayed different combinations of resistances to antibiotics belonging to the following families: penicillins (ampicillin), cephalosporins (cefotaxime), aminoglycosides (streptomycin and kanamycin), tetracyclines, folate inhibitors (sulfonamides and trimethoprim), and fluoroquinolones (ciprofloxacin). The resistance phenotypes and responsible genes are shown in Table 1, and their genetic context will be detailed below.

### 3.3. The IncHI2-ST1 Plasmids Harbor Multiple Resistance Genes

In all isolates, the *bla*_CTX-M-9_ gene responsible for cefotaxime resistance was located on large IncHI2-ST1 plasmids, with sizes ranging between 256–350 kb (Table 1). Many other resistance genes and multiple genetic elements potentially involved in DNA mobility were also carried by these plasmids. The highest number of resistance determinants was detected in the IncHI2-ST1 plasmid of HUD 1/16 (pHUD 1/16), whose entire nucleotide sequence was determined. This plasmid was nearly identical to p123 from *S. enterica* strain S17BD06931 of unreported serovar (accession number CP099763) and closely related to plasmids found in several strains of monophasic *S*. Typhimurium (CP082418, OU015718, OU015720, OU015717, and MK191844), biphasic *S*. Typhimurium (KX810825, LK056646, and CP021463), as well as in other *S. enterica* serovars, including *S*. Agona, *S*. Brandenburg, *S*. Concord, *S*. Infantis, *S*. Newport and *S*. Senftenberg (CP071389, CP082465, CP028197, LN555650, CP012599, and CP039271, respectively). In all these cases, the identity was higher than 99.9%, with more than 80% coverage.

**Figure 1:**
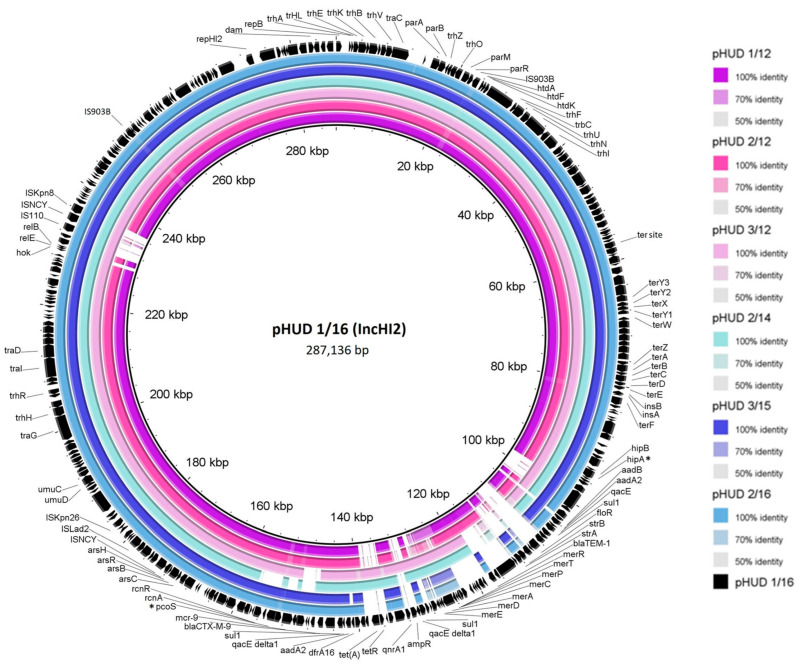
BRIG (Blast Ring Image Generator) comparison of *bla*_CTX-M-9_-bearing IncHI2-ST1 plasmids found in monophasic *S*. Typhimurium ST34 isolates from a Spanish hospital. Each ring corresponds to a plasmid according to the color code specified on the right-hand side. pHUD 1/16 was entirely sequenced in this study and used as the reference (inner black ring). Relevant genes of this plasmid are displayed in the outer black ring, represented by arrows indicating the direction of transcription. Note that only the resistance genes are annotated for clarity in the large resistance region located between the *hipA* and *pcoS* genes (marked with an asterisk) (see Figure 2 for further details). To generate the image, the concatenated contigs of the other plasmids, as identified by PLACNETw, were used. Thus, the presence/absence of genes, but not necessarily the synteny, is accurate. Also, genes carried by these plasmids but absent in the control are excluded from the comparison but included in Figure 2.

A BRIG comparison of pHUD 1/16 with all other IncHI2-ST1 plasmids under study is shown in Figure 1. Apart from genes involved in plasmid replication and maintenance, all contained apparently complete sets of genes for conjugative transfer (*trh* and *tra*), distributed into two separate regions. In agreement with this, all plasmids proved to be self-transferable at 28 °C but not at 37 °C, as expected for the IncHI2 group (Table S4) [25]. For all but one plasmid (pHUD 3/12), the conjugation frequencies ranged between 1.3 × 10^−2^ and 2.6 × 10^−4^ transconjugants per donor cell. These values align with data reported for pR478 [25], the prototype IncHI2-ST1 plasmid first isolated from *Serratia marcescens* in 1969 [26]. The frequency of conjugation of pHUD 3/12 was remarkably lower (1.7 × 10^−7^ transconjugants per donor cell), but the reason for this remains unknown. In all cases, the resistance phenotypes of the transconjugants coincided with those associated with each of the IncHI2-ST1 plasmids (see Table 1), and the presence of the IncHI2 replicon was demonstrated by PBRT (not shown). Consistent with horizontal transfer, the plasmids under study were closely related, with the number of SNP differences between them ranging from 0–50 (Table S3).

Apart from genes involved in basic biological functions of IncHI2 plasmids, genes for resistance to tellurium (also separated in two regions: *terZABCDEF*, with IS*1* inserted within the intergenic region between *terE* and *terF*, and *terY3Y2XY3W*) and to arsenic compounds (*arsCBRH*) were found in the plasmids under study. These genes are commonly carried by the IncHI2 group and were already present in pR478 [26]. In contrast, they lacked most of the *pco* genes for copper resistance, which are also typical for this group.

All other resistance genes of pHUD 1/16 clustered within a large DNA segment of approximately 56 kb, located between the toxin-antitoxin genes *hipBA* and the copper resistance gene *pcoS*. A nearly identical region, including all antibiotic resistance genes, was only shared by plasmid p123 of *S. enterica* strain S17BD06931 (see above). However, closely related arrangements were present in plasmids found in several *Enterobacter* species, such as *E. cloacae* (CP052871; AP022134), *E. hormaechei* (CP053645) and *E. roggenkampii* (CP083854), with a shared identity of ≥99.98 and 82% coverage.

**Figure 2:**
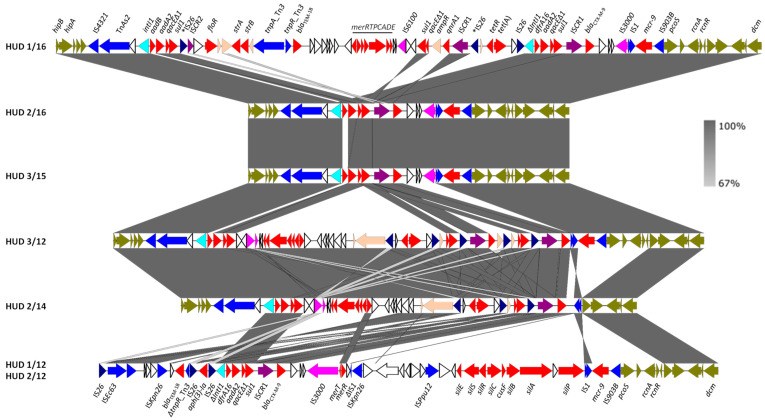
Detailed structure and comparison of the resistance clusters found in the IncHI2-ST1 plasmids of monophasic *S*. Typhimurium ST34 isolates. Protein coding regions (ORFs) are represented by arrows indicating the direction of transcription and colored according to their function: red, resistance; light blue, DNA mobility, with IS*26* highlighted in dark blue, IS*3000* and IS*6000* in light purple, and IS*CR* elements in dark purple; orange, other roles; olive green, plasmid ORFs flanking the resistance clusters. The two copies of IS*26,* flanked by direct repeats that delineate a fragment that was probably inverted in pHUD 1/16, are marked with asterisks. The alignments were created with EasyFig BLASTn. The gray shading between regions reflects nucleotide sequence identities according to the scale on the figure’s right.

The genetic structure of this segment and its comparison with the assembled resistance clusters from the remaining isolates is shown in Figure 2. The *bla*_CTX-M-9_ gene was invariably located immediately downstream of IS*CR1,* and in all except two of the plasmids (pHUD 3/12 and pHUD 2/14), IS*CR1*-*bla*_CTX-M-9_ resided within defective In60-like complex class 1 integrons, with the class 1 integrase encoding *intI1* gene, being either intact or truncated by insertion of IS*26.* In the variable region of these integrons, two different gene cassette arrays were found: (i) *dfrA16*-*aadA2b*, the array typical for In60 itself [27], was present in the plasmids of four isolates (pHUD 1/12, pHUD 2/12, pHUD 1/16 and pHUD 2/16); and (ii) *aadB* [*ant(2″)-Ia*]-*aadA2b* [28], which resided in a single plasmid (pHUD 3/15). In these integrons, the 3′-conserved segment (3′-CS) carrying the *qacEΔ1*-*sul1* genes was located upstream of IS*CR1*, and the insertion sequence IS*3000* (intact or truncated by IS*1*) was placed downstream of *bla*_CTX-M-9_, as previously reported for In60 [28]. However, they lacked the second copy of 3′-CS, which is typically located downstream of IS*3000* in complex In60-like integrons. In pHUD 3/12 and pHUD 2/14, IS*CR1*-*bla*_CTX-M-9_ could not be unambiguously associated with other components of the In60-like integrons. These results underline the high diversity amongst In60-like integrons acting as vehicles of *bla*_CTX-M-9_, as reported previously [29].

CTX-M-9 is one of the most common ESBLs produced by *S. enterica*. This is mainly due to the spread of a particular clone of *S*. Virchow, with phage type 19 and positive for In60. This clone has been circulating in Southern Europe (Spain and France), both in humans and food-producing animals, mostly poultry [30,31,32,33,34]. Like in isolates reported herein, the integron was located on large plasmids (335 kb and 370 kb), either assigned to IncHI2 or not experimentally typed at the time [31,32]. In60-like integrons positive for *bla*_CTX-M-9_ were also found in other *S. enterica* serovars, including *S*. Bovismorbificans, where the *aadB*-*aadA2b* variant was first detected [28].

Interestingly, the IncHI2-ST1 plasmids of two isolates (HUD 3/12 and HUD 1/16) also harbored *qnrA1*, which was associated with a MIC to ciprofloxacin of 0.5 mg/L in both, and with IZD (inhibition zone diameters) for pefloxacin of 19 and 18 mm, respectively. According to EUCAST breakpoints, these values are connected with therapeutic failure. In both isolates, *qnrA1* was linked to IS*CR1* as part of complex class 1 integrons related to In36 [35]. The In36-like integron of pHUD 1/16 differs from prototype In36 by the variable region (*aadB*-*aadA2* instead of *dfrA16*-*aadA2*) and the inversion of a large segment containing part of the integron, including IS*CR1* and *qnrA1*, which was probably mediated by IS*26*. The latter mechanism, supported by the presence of 8 bp direct repeats found in opposite orientations (ATAAAACG/CGTTTTAT; Figure 2), could have resulted from intramolecular IS*26* transposition by the *trans* pathway [36]. In this scenario, the non-inverted element would have included the 3′-CS duplication typical of complex class 1 integrons, which was nevertheless absent in the In60-like integrons bearing *bla*_CTX-M-9_ (see above). Similarly, although all In36 components could be identified in the resistance cluster of pHUD 3/12 (i.e., the integrase gene, the variable *dfrA16*-*aadA2* region, the insertion sequence IS*6100*, and the two copies of 3′-CS), the order of these elements was substantially altered, probably due to the activity of IS*26*.

In addition to the resistance genes provided by complex class 1 integrons bearing IS*CR1*, six out of the seven IncHI2-ST1 plasmids carried the *mcr-9* gene. The latter was invariably flanked by the insertion sequences IS*1* and IS*903B* (belonging to the IS*5* family), located adjacent to *pcoS* (Figure 2). The *qseC*/*qseB* genes, reported downstream of *mcr-9* in several Enterobacterales [37], were not found at this position in our isolates but were nevertheless present in the bacterial chromosome. These genes encode a two-component regulatory system shown to be involved in the induction of colistin resistance by subinhibitory concentrations of the antibiotic [37]. So, although the isolates carrying *mcr-9* in the present study were susceptible to colistin, with MIC_S_ of 1 or 2 mg/L, the possible induction of resistance cannot be ruled out. Originally reported in 2019 in a clinical isolate of *S.* Typhimurium from the USA, which was also susceptible to colistin [38], *mcr-9* has now been identified in 21 countries across the world, particularly in members of the order *Enterobacterales* [39,40]. In Spain, it has already been circulating in monophasic isolates of *S*. Typhimurium at least since 2012 (present study) and in members of the *E. cloacae* complex at least since 2016 [41].

As evident from Figure 2, some of the resistance clusters of the IncHI2-ST1 plasmids under study carried additional antibiotic resistance genes, such as *bla*_TEM-1B_ (ampicillin), *floR* (chloramphenicol/florfenicol), *aph(3′)-1a* (kanamycin), *strA*-*strB* (streptomycin), and *tet*(A) (tetracycline). The *floR* gene, found in pHUD 1/16, was associated with a truncated version of IS*CR2*, another IS*91*-like element originally reported in STX, a chromosomal ICE of the STX/R391 family conferring multidrug resistance to *Vibrio cholerae* [8,42]. Genes for resistance to heavy metals, including *merRTPCADE* (mercury) and *silESRCcusFsilBAP* (silver/copper), were also part of the clusters. Like the *ter* and *ars* genes mentioned above, these genes were already present in the prototype pR478 of *S. marcescens* [27].

Finally, regarding their location, the resistance clusters of four of the seven IncHI2-ST1 plasmids were placed between the *hipA* (for a toxin-antitoxin system) and the *pcoS* genes. As shown in Figure 1, the clusters could then be extended to incorporate the *ter* and *ars* genes located on either side. In two other plasmids, pHUD 1/12 and pHUD 2/12, which share the same resistance cluster, only the right border could be identified with *pcoS*, whereas the left border stayed elusive. In the remaining plasmid, pHUD 2/14, which was the only one lacking *mcr-9*, a deletion removing this gene also affected the adjacent *pcoS* gene. Accordingly, the resistance cluster of this plasmid was placed between *hipA* and a gene encoding a hypothetical protein adjacent to *pcoS* in the other IncHI2-ST1 plasmids (Figure 2).

### 3.4. Chromosomal Regions Encoding Resistance to Antibiotics and Heavy Metals

As indicated, SGI-4 and RR, both of chromosomal location, are typically associated with the monophasic variant of *S*. Typhimurium ST34. The genetic structures of both elements in the isolates under study are described in the following sections.

#### 3.4.1. The Genomic Island SGI-4

The ICE SGI-4 was detected in five out of the seven isolates under study (71.4%). The island was highly conserved, sharing more than 99% identity between the isolates and also with genomic islands found in other ST34 monophasic strains, available in the literature and/or deposited in databases. The island was inserted between chromosomal genes encoding transcriptional regulators of the *merR* and *tetR* (*yjdC*) families and consisted of 80,795 bp flanked by perfect direct repeats of 55 bp (the *attL* and *attR* sites). SGI4 contains genes associated with mobility (integration, excision, and conjugation) of the ICE, as well as clusters of genes, *arsRSD2A2BCA1D1*, *silESRCFBAGP*, and *pcoGE1ABCDRSE2*, conferring resistance to arsenic, silver and copper compounds (see [13]). It has been proposed that the increased use of heavy metals as growth promoters in pork production, particularly after the banning of antibiotics in the EU since 2006 with such an aim, could have contributed to the epidemiological success of the bacteria with SGI-4 [14,43].

However, two of the HUD isolates (HUD 3/12 and HUD 2/14) lacked SGI-4. The sporadic absence of the island has previously been reported and was attributed to its elimination from the chromosome through site-specific recombination between the *att* sites. This would regenerate the original insertion site, which was in fact observed in the isolates.

#### 3.4.2. The RR Resistance Regions and the Genetic Bases of the Monophasic Phenotype

**Figure 3:**
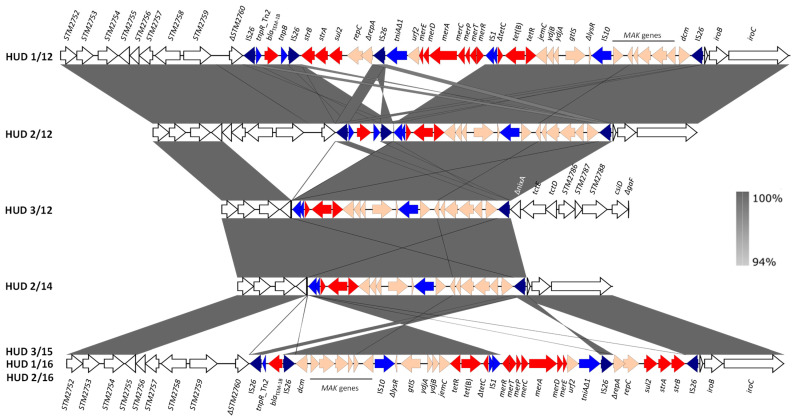
Genetic organization and comparisons of the chromosomal RR regions found in the monophasic *S*. Typhimurium ST34 isolates from a Spanish hospital. Coding regions are represented by arrows indicating the direction of transcription and colored according to their function: red, resistance; blue, DNA mobility, with IS*26* highlighted in darker blue; orange, other roles; white, flanking chromosomal ORFs named according to accession number NC_003197, which corresponds to the chromosome of *S*. Typhimurium LT2 [45]. The alignments were created with EasyFig BLASTn. The gray shading between regions reflects nucleotide sequence identities according to the scale in the right bar.

In contrast to the conservation of SGI-4, the RR regions displayed a high variability with four distinct structures shown by the seven isolates (Figure 3). In all cases, the RR element consisted of a contiguous module, as originally reported for monophasic *S*. Typhimurium strain 00-2006 [18]. However, multiple differences were observed, affecting the RR itself and the insertion site into the bacterial chromosome (Figure 3). Specific variations involved: (i) the genes responsible for the tetra-resistance phenotype, with *bla*_TEM-1B_, *strA*-*strB*, *sul2* and *tet*(B), *bla*_TEM-1B_ and *tet*(B), and only *tet*(B) in four, one and two isolates, respectively (see also Table 1); (ii) the *mer* locus, absent in three isolates; (iii) the number of IS*26* copies, with four, three and one, in four, one and two isolates, respectively; (iv) the orientation of the RR region, most of which was inverted in three isolates sharing the same structure (HUD 3/15, HUD 1/16 and HUD 2/16). In agreement with the monophasic phenotype, the presence of RR was associated with large chromosomal deletions, always removing the *fljA*, *fljB,* and *hin* genes, whose products are responsible for the expression of the second phase flagellin and phase variation [44]. In five isolates, the RR element occupied the same position within the bacterial chromosome, between ΔSTM2760 and Δ*hp*-*iroB*-*iroC*, accounting for a deletion of ca. 15 kb. In the remaining isolates, larger deletions occurred, encompassing from ΔSTM2758 to Δ*hp*-*iroB*-*iroC* (22 kb; HUD 2/14) or from ΔSTM2758 up to Δ*nixA* (STM2783; 37 kb; HUD 3/12) (Figure 3).

## 4. Conclusions

WGS analysis permitted us to determine the genetic bases of multidrug resistance in clinical isolates belonging to the emerging monophasic ST34 variant of *S*. Typhimurium. The obtained results highlight the critical role of IncHI2-ST1 conjugative plasmids in disseminating resistances to broad-spectrum cephalosporins and other antibiotics with outmost importance in human medicine as to heavy metals. Our results reveal the ongoing evolution of these highly variable plasmids, which is owed to a wealth of genetic elements involved in DNA mobility. In fact, most resistance genes were clustered within a region of great plasticity crowded by IS*CR* elements, usually associated with complex class 1 integrons and many other transposable elements. Consequently, a wide range of resistance phenotypes was observed, which allows bacterial adaptation under the selective pressure exerted by exposure to antibiotics and biocides.

## Figures and Tables

**Table 1:** Origin, resistance phenotype and genetic basis of antibiotic resistance in monophasic *S*. Typhimurium ST34 isolates from a Spanish hospital.

Isolate ^a^	Patient Sex/Age ^b^	Resistance Phenotype ^c^/ Antibiotic Resistance Genes ^d^	Chromosome Plasmids ^e^ (Size bp)
HUD 1/12	M/5	AMP, CTX, KAN, STR, SUL, TET, TMP/ *bla*_TEM-1B_, *strA*, *strB*, *sul2*, *tet*(B) *bla*_TEM-1B_, *bla*_CTX-M-9_, *aph(3′)-Ia*, *aadA2b*, *sul1, dfrA16*, *mcr-9* - -	Chr (4,970,905) IncHI2-ST1 (349,612) unk * (4072) ColE * (3830)
HUD 2/12	F/10	AMP, CTX, KAN, STR, SUL, TET, TMP/ *bla*_TEM-1B_, *tet*(B) *bla*_TEM-1B_, *bla*_CTX-M-9_, *aph(3′)-1a*, *aadA2b*, *sul1*, *dfrA16*, *mcr-9* -	Chr (4,993,648) IncHI2-ST1 (301,830) ColE * (3820)
HUD 3/12	M/9	AMP, CTX, CIP, STR, SUL, TET, TMP/ *tet*(B) *bla*_CTX-M-9_, *qnrA1*, *aadA2b*, *sul1, tet*(A), *dfrA16, mcr-9*	Chr (4,845,756) IncHI2-ST1 (270,777)
HUD 2/14	F/78	AMP, CTX, STR, SUL, TET, TMP/ *tet*(B) *bla*_CTX-M-9_, *aadA2b*, *sul1*, *tet*(A), *dfrA16*	Chr (4,862,133) IncHI2-ST1 (263,144)
HUD 3/15	M/3	AMP, CTX, STR, SUL, TET/ *bla*_TEM-1B_, *strA*, *strB*, *sul2*, *tet*(B) *bla*_CTX-M-9_, *aadB*-*aadA2b*, *sul1, mcr-9*	Chr (4,974,577) IncHI2-ST1 ^f^ (308,586)
HUD 1/16	F/9	AMP, CTX, CHL, CIP, STR, SUL, TET, TMP/ *bla*_TEM-1B_, *strA, strB*, *sul2*, *tet*(B) *bla*_TEM-1B_, *bla*_CTX-M-9_, *floR*, *qnrA1*, *aadB-aadA2b*, *strA*, *strB*, *sul1*, *tet*(A), *dfrA16*, *mcr-9*	Chr (4,986,724) IncHI2-ST1 * (287,136)
HUD 2/16	M/8	AMP, CTX, STR, SUL, TET, TMP/ *bla*_TEM-1B_, *strA*, *strB*, *sul2*, *tet*(B) *bla*_CTX-M-9_, *aadA2b*, *sul1, dfrA16, mcr-9*	Chr (4,962,945) IncHI2-ST1 (255,829)

^a^ The isolates were collected from clinical samples (feces) analyzed at the “Hospital Universitario de Donostia,” San Sebastian, Basque Country, Spain, and designated by the initials of the hospital (HUD) followed by a serial number/year of isolation (e.g., 12 referring to 2012). Their antigenic formula, determined in silico by SeqSero (https://cge.cbs.dtu.dk/services/, last accessed on 16 February 2023), was 4:i:-. ^b^ M, male; F, female. ^c^ AMP, ampicillin; CTX, cefotaxime; CHL, chloramphenicol; CIP, ciprofloxacin; KAN, kanamycin; STR, streptomycin; SUL, sulfonamides; TET, tetracycline; TMP, trimethoprim; -, no resistance gene(s) located on the corresponding plasmids. ^d^ The *mcr-9* gene, found in all but one isolate, did not confer colistin resistance. The *aac(6′)-Iaa* gene, cryptic in *Salmonella*, was detected in the chromosome of all isolates. ^e^ Inc, plasmid incompatibility group; unk, unknown; *, circularized plasmid. ^f^ The *smr0018* locus identified by pDLST was duplicated in the IncHI2 plasmid of this isolate.

## Data Availability

The genome sequences generated in the present study were deposited in GenBank under accession numbers JAICCN000000000, JAICCM000000000, JAICCL000000000, JAICCK000000000, JAICCJ000000000, JAICCI000000000, and JAICCH000000000 for HUD 1/2, HUD 2/12, HUD 3/12, HUD 2/14, HUD 3/15, HUD 1/16 and HUD 2/16, respectively.

## Supplementary Materials

The following supporting information can be downloaded at: https://www.mdpi.com/article/10.3390/antibiotics12030547/s1, Table S1: Accession numbers of the genomes of monophasic *Salmonella enterica* serovar Typhimurium ST34 isolates, positive for *bla*_CTX-M-9_, from a Spanish hospital; parameters related to the quality of the assemblies as provided by VelvetOptimizer implemented in PLACNET (https://ormictools.com/placnet-tool). Table S2: Oligonucleotides used for the assembly of pHUD 1/16, the resistance regions of other IncHI2-ST1 plasmids, and the chromosomal RR regions found in monophasic *Salmonella enterica* serovar Typhimurium ST34 isolates from a Spanish hospital (References [46,47] are cited in supplementary materials). Table S3: SNP differences between monophasic *Salmonella enterica* serovar Typhimurium ST34 isolates from a Spanish hospital (HUD; upper similarity matrix) and between their IncHI2-ST1 plasmids (pHUD; lower similarity matrix). Table S4: Conjugation frequencies of IncHI2-ST1 plasmids found in monophasic *Salmonella enterica* serovar Typhimurium ST34 isolates from a Spanish hospital.
