# Supplementary material for: Spread of blaCTX-M-9 and Other Clinically Relevant Resistance Genes, Such as mcr-9 and qnrA1, Driven by IncHI2-ST1 Plasmids in Clinical Isolates of Monophasic Salmonella enterica Serovar Typhimurium ST34
Source: Antibiotics (Basel). 2023 Mar 9;12(3):547. doi: 10.3390/antibiotics12030547 (PMC10044134; doi:10.3390/antibiotics12030547)
Supplement: Supplementary file 1 [file antibiotics-12-00547-s001.zip › antibiotics-2223653-supplementary.pdf]

*Supplementary material*

# Spread of *bla*$_{CTX-M-9}$ and other clinically relevant resistance genes, such as *mcr-9* and *qnrA1*, driven by IncHI2-ST1 plasmids in clinical isolates of monophasic *Salmonella enterica* serovar Typhimurium ST34

**Table S1.** Accession numbers of the genomes of monophasic *Salmonella enterica* serovar Typhimurium ST34 isolates, positive for *bla*$_{CTX-M-9}$, from a Spanish hospital; parameters related to the quality of the assemblies as provided by VelvetOptimizer implemented in PLACNET (https://castillo.dicom.unican.es/upload/).

| Isolate[a] | kmer | Contigs | N50 | Longest contig (bp) | Total bp in contigs | Contigs > 1 kb | Library | Coverage | GenBank accession number |
|---|---|---|---|---|---|---|---|---|---|
| HUD 1/12 | 117 | 131 | 319,261 | 1,014,100 | 5,323,913 | 52 | 528+/-145 | 30x | JAICCN000000000 |
| HUD 2/12 | 117 | 123 | 415,385 | 929,792 | 5,309,364 | 44 | 546+/-147 | 35x | JAICCM000000000 |
| HUD 3/12 | 117 | 104 | 341,666 | 1,012,649 | 5,122,035 | 36 | 539+/-148 | 21x | JAICCL000000000 |
| HUD 2/14 | 115 | 96 | 341,611 | 929,895 | 5,130,018 | 32 | 561+/-152 | 25x | JAICCK000000000 |
| HUD 3/15 | 119 | 98 | 415,425 | 838,161 | 5,288,026 | 34 | 561+/-149 | 25x | JAICCJ000000000 |
| HUD 1/16 | 117 | 126 | 319,361 | 650,392 | 5,256,219 | 46 | 539+/-145 | 24x | JAICCI000000000 |
| HUD 2/16 | 89 | 112 | 341,443 | 650,087 | 5,222,712 | 39 | 569+/-152 | 25x | JAICCH000000000 |

[a], Isolates are designated with the initials of the center which supplied them, followed by a serial number/last two numbers of the year of recovery. HUD, "Hospital Universitario de Donostia", Basque Country, Spain.

**Table S2.** Oligonucleotides used for the assembly of pHUD 1/16, the resistance regions of other IncHI2-ST1 plasmids, and the chromosomal RR regions found in monophasic *Salmonella enterica* serovar Typhimurium ST34 isolates from a Spanish hospital.

| Primer name | Sequence (5′-3′) |
|---|---|
| hp | AGATTCAGCCTGATCGCAGATTG |
| Tn3 fam.1 | GTCGGTTTCGTTGAACGTGTAG |
| intI1.1 | TCGCCATCACATGTATGACCAG |
| intI1.2 | CTGGTCATACATGTGATGGCGA |
| intI1.3 | GAGCAGCAACGATGTTACGCAG |
| aadB1 | GAGTTACTTGACTGCGAACCTG |
| aadB2 | CAGGTTCGCAGTCAAGTAACTC |
| sul1.1 | TGCTAGGCATGATCTAACCCTC |
| sul1.2 | GAGGGTTAGATCATGCCTAGCA |
| qacE delta1 | CTCACAGCCAAACTATCAGGTC |
| aadA2.1 | GACGCTTAGCACCTCTGATAGT |
| aadA2.2 | ACTATCAGAGGTGCTAAGCGTC |
| floR.1 | ATAGAGGCTCAACGTGAGTTGG |
| floR-2 | CCAACTCACGTTGAGCCTCTAT |
| ISVsa3 | TGAAGTACTGCCTGAACAACCC |
| tnpR.1 | TGCTCATCAGCTCAGTATTGCC |
| blaTEM1.1 | AGACAGATCGCTGAGATAGGTG |
| hp | ACAGCTTTCTAAGAACGGTGGG |
| merE | CCTTCAAGGGAAGATCATGACC |
| merR.fl | CAAAGGGGTAAGTGACCAAACC |
| IS6100.1 | TGCAGCGATCACCGAATTGAAG |
| IS6100.2 | TGCAGCGATCACCGAATTGAAG |
| GNAT.1 | CTTCATCGCAGAACGCCTAGAA |
| GNAT.2 | TTCTAGGCGTTCTGCGATGAAG |
| **Primer name** | **Sequence (5′-3′)** |
| hypA | TGCAGTGCACTCTCCTCAACAT |
| ampR.1 | CACTCCAGAGTATCCGCAATCC |
| ampR.2 | GAGGTATCGAACACCATTACCG |

| | |
|---|---|
| qnrA1.2 | GCTTTGGCATAGAGTTCAGGGA |
| qnrA1.3 | TCCCTGAACTCTATGCCAAAGC |
| ISCR1.1 | CTCACGAGTTGTTATCAGCCAC |
| ISCR1.2 | TCCAACACTGCTCAACACTGCC |
| tetA fl | GTGAATCTGCTATGGCAATCGG |
| tetR fl | AGGCTGTTTGCTTCTGACGAGA |
| drfA16.2 | ATCGAGCGAGATGGAGACATAG |
| drfA16.3 | TGTCTCCATCTCGCTCGATGTC |
| CTXM9.1 | CACCACCAATGATATTGCGGTG |
| CTXM9.2 | TGGTACTGCACATTGGAAAGCG |
| IS3000.1 | GTGGATCACATCTTCCTGGATG |
| IS3000.2 | CTTCAACTCCAGGATACTCAGC |
| mcr9.1 | TTGCCAGAAGTAAAACCAGCGG |
| mcr9.2 | CCGCTGGTTTTACTTCTGGCAA |
| mcr9.3 | GAATGTGCACGTAGCGATATCG |
| IS5 fam.1 | AGTGGCTAATCAGCGAATGACC |
| IS5 fam.2 | AGTGGCTAATCAGCGAATGACC |
| IS5 fam.3 | GGTCATTCGCTGATTAGCCACT |
| pcoS | GCCATTAAGGGTGCTAATCAGG |
| PPR | CCATTGAATGGTCAACCCCTAC |
| IS90-3B fl | AGCTTCTCCCTTCCCTTGACAC |
| traC fl | TATGCTCAAGAAGTAGCAGCGG |
| parA fl | ACAATCTTCCGCTCGTACGATG |
| parM | CGCAGTTCGCATTATCTTTGGC |
| htdF fl | ACAGATTCAGCTGAAGCCTGAG |
| terD | TCTGTCTGAAGATGCCTCAACC |
| insB IS1 | TGACTTTGTCATGCAGCTCCAC |
| insA IS1 | GAACAGGAGGGACAGCTGATAG |
| Tn3 fam.2 | CAATCGCTTCAGTCTGAACGAC |
| Tn3 fam.3 | GTCGTTCAGACTGAAGCGATTG |
| aph (3´)-Ia.1 | ACCATGAGTGACGACTGAATCC |
| aph (3´)-Ia.2 | GGATTCAGTCGTCACTCATGGT |

| | |
|---|---|
| IS5 fam fl | TGAGTCCGATAGTGGCAATAGC |
| silS | GTCCGGAATGAAGATGGAAACG |
| silP | GCCTGCTTTATCCTGTGTATGG |
| silE | AACCAAAGCCCAGCAAAGATGC |
| STM2760 | CATCTCATTGACAAGCCGGATC |
| STM2755 | AGATCATATCCACCACCACCTG |
| iroB-R | ATAGCAGTCCATACAGTGGTGG |
| nixA | ATGGTTCGTTGATTGGTACGGC |
| dcm | CTTCTATCGATGCATCCATCCC |
| repA | ATGTTATCGACCAGTACCGAGC |
| tetC | AGGCATCACTTCTTGGATAGGG |
| merR | ATGGAAACCGTGCTGTCTGAAC |
| urf2-F[46] | GGAGATCGAACTGACCGAATCGG |
| strB-R[46] | GGATCGTAGAACATATTGGC |
| strB-F[46] | ATCGTCAAGGGATTGAAACC |
| strA-R[46] | CCAATCGCAGATAGAAGGC |
| strA-F[46] | CTTGGTGATAACGGCAATTC |
| blaTEM-F[47] | TTGGGTGCACGAGTGGGT |
| blaTEM-R[47] | TAATTGTTGCCGGGAAGC |

46. Martínez, N.; Mendoza, M.C.; Guerra, B.; Gonzalez-Hevia, M.A.; Rodicio, M.R. Genetic basis of antimicrobial drug resistance in clinical isolates of *Salmonella enterica* serotype Hadar from a Spanish region. *Microb Drug Resist* **2005**, *11*, 185-193.

47 Arlet, G.; A. Philippon, A. Construction by polymerase chain reaction and use of intragenic DNA probes for three main types of transferable betalactamases (TEM, SHV, CARB). *FEMS Microbiol. Lett.* **1991**, *66*, 19-25.

**Table S3.** SNP differences between monophasic *Salmonella enterica* serovar Typhimurium ST34 isolates from a Spanish hospital (HUD; upper similarity matrix), and between their IncHI2-ST1 plasmids (pHUD; lower similarity matrix).

| | HUD 1/12 | HUD 1/16 | HUD 2/12 | HUD 2/14 | HUD 2/16 | HUD 3/12 | HUD 3/15 |
|---|---|---|---|---|---|---|---|
| **HUD 1/12** | 0 | 67 | 5 | 401 | 77 | 395 | 76 |
| **HUD 1/16** | 67 | 0 | 72 | 372 | 14 | 366 | 11 |
| **HUD 2/12** | 5 | 72 | 0 | 406 | 78 | 400 | 77 |
| **HUD 2/14** | 401 | 372 | 406 | 0 | 384 | 16 | 381 |
| **HUD 2/16** | 77 | 14 | 78 | 384 | 0 | 378 | 3 |
| **HUD 3/12** | 395 | 366 | 400 | 16 | 378 | 0 | 375 |
| **HUD 3/15** | 76 | 11 | 77 | 381 | 3 | 375 | 0 |

min: 3 max: 406

| | pHUD 1/16_1 | pHUD 1/12_1 | pHUD 2/12_1 | pHUD 2/14_1 | pHUD 2/16_1 | pHUD 3/12_1 | pHUD 3/15_1 |
|---|---|---|---|---|---|---|---|
| **pHUD 1/16_1** | 0 | 40 | 44 | 9 | 10 | 9 | 10 |
| **pHUD 1/12_1** | 40 | 0 | 4 | 41 | 50 | 41 | 50 |
| **pHUD 2/12_1** | 44 | 4 | 0 | 45 | 50 | 45 | 50 |
| **pHUD 2/14_1** | 9 | 41 | 45 | 0 | 19 | 0 | 19 |
| **pHUD 2/16_1** | 10 | 50 | 50 | 19 | 0 | 19 | 0 |
| **pHUD 3/12_1** | 9 | 41 | 45 | 0 | 19 | 0 | 19 |
| **pHUD 3/15_1** | 10 | 50 | 50 | 19 | 0 | 19 | 0 |

min: 0 max: 50

**Tabla S4.** Conjugation frequencies of IncHI2-ST1 plasmids found in monophasic *Salmonella enterica* serovar Typhimurium ST34 isolates from a Spanish hospital.

| **Donor strain** [a] | **Transfer frequency** [b] **(standard deviation)** | |
|---|---|---|
| | **28 °C** | **37 °C** |
| HUD 1/12 | $1.45 \times 10^{-3}$ ($0.27 \times 10^{-3}$) | $\leq 2.2 \times 10^{-10}$ |
| HUD 2/12 | $1.35 \times 10^{-3}$ ($0.06 \times 10^{-3}$) | $\leq 1.4 \times 10^{-10}$ |
| HUD 3/12 | $1.70 \times 10^{-7}$ ($0.02 \times 10^{-7}$) | $\leq 1.6 \times 10^{-10}$ |
| HUD 2/14 | $1.27 \times 10^{-2}$ ($0.69 \times 10^{-2}$) | $\leq 3.2 \times 10^{-9}$ |
| HUD 3/15 | $2.61 \times 10^{-4}$ ($0.99 \times 10^{-4}$) | $\leq 3.4 \times 10^{-8}$ |
| HUD 1/16 | $3.10 \times 10^{-3}$ ($1.10 \times 10^{-3}$) | $\leq 1.1 \times 10^{-10}$ |
| HUD 2/16 | $2.31 \times 10^{-3}$ ($0.91 \times 10^{-3}$) | $\leq 5.8 \times 10^{-9}$ |

[a], HUD, "Hospital Universitario de Donostia", San Sebastian, Basque Country, Spain. [b], Conjugation frequencies are reported as the number of transconjugants per donor cell, and result from the average of two independent experiments. Selection was performed with rifampicin (recipient strain) and cefotaxime (IncHI2-ST1 plasmids).
